# Efficacy and safety of sacubitril-valsartan in patients with heart failure: a systematic review and meta-analysis of randomized clinical trials: A PRISMA-compliant article

**DOI:** 10.1097/MD.0000000000028231

**Published:** 2021-12-30

**Authors:** Jiezhong Lin, Jianyi Zhou, Guiting Xie, Jinguang Liu

**Affiliations:** Department of Cardiovascular Medicine, Huizhou Municipal Center Hospital, Huizhou, Guangdong, China.

**Keywords:** efficacy, heart failure, meta-analysis, randomized clinical trials, sacubitril-valsartan, safety

## Abstract

**Background:**

To investigate the efficacy and safety of sacubitril-valsartan in patients with heart failure, relevant randomized clinical trials (RCTs) were analyzed.

**Methods:**

We used Cochrane Library, PubMed web of science, CNKI, VIP, Medline, ISI Web of Science, CBMdisc, and Wanfang database to conduct a systematic literature research. A fixed-effects model was used to evaluate the standardized mean differences (SMDs) with 95% confidence intervals. We conducted sensitivity analysis and analyzed publication bias to comprehensively estimate the efficacy and safety of sacubitril-valsartan in patients with heart failure.

**Results:**

Among 132 retrieved studies, 5 relevant RCTs were included in the meta-analysis. The result showed that left ventricular ejection fraction (LVEF) was improved after sacubitril-valsartan in patients with heart failure, with an SMD (95% CI of 1.1 [1.01, 1.19] and *P* < .00001 fixed-effects model). Combined outcome indicators showed that, combined outcome indicators showed that, compared with control group, the left ventricular volume index (LAVI) (WMD = −2.18, 95% CI [−3.63, −0.74], *P* = .003), the E/e’ (WMD = −1.01, 95% CI [−1.89, −0.12], *P* = .03), the cardiovascular death (RR = 0.89, 95% CI [0.83, 0.96], *P* = .003], and the rehospitalization rate of heart failure (RR = 0.83, 95% CI [0.78, 0.88], *P* < .01) decreased more significantly, but it had no effect on renal function (WMD = 0.74, 95% CI [0.54, 1.01], *P* = .06).

**Conclusions:**

The present meta-analysis suggested that sacubitril-valsartan may improve the cardiac function of heart failure. Given the limited number of included studies, additional large sample-size RCTs are required to determine the long-term effect of cardiac function of sacubitril-valsartan in patients with heart failure.

## 1 Introduction

Heart failure (HF) is a clinical syndrome of ventricular filling and/or impaired ejection function caused by various cardiac structural or functional diseases. It is the end stage of various cardiovascular diseases and it is known as the “last battlefield” of cardiovascular diseases.[^1^,^2^] According to the epidemiological analysis reported, the prevalence rate of heart failure (HF) in the global population is 0.9%, and the prevalence rate increases significantly.[^3^,^4^] Moreover, the prevalence of HF is increasing, which brings a very heavy economic burden to our country.^[5]^ Several drugs have been applied to heart failure, such as β blockers, calcium-channel blockers, angiotensin-converting enzyme (ACE) inhibitors, and angiotensin receptor blockers (ARBs),[^6^,^7^,^8^,^9^] but there was no obvious efficacy.

Sacubitril–valsartan is an angiotensin receptor–neprilysin inhibitor which applied to treat that heart failure.^[10]^ Neprilysin degrades biologically active natriuretic peptides, including atrial natriuretic peptide (ANP), B-type natriuretic peptide (BNP), and C-type natriuretic peptide, but not the biologically inert NT-proBNP, which is not a substrate for this enzyme.^[11]^ In the PARADIGM-HF (Prospective Comparison of ARNI with ACEI to Determine Impact on Global Mortality and Morbidity in Heart Failure) trial,[^12^,^13^] the use of sacubitril–valsartan resulted in a lower risk of death for heart failure than enalapril in this population. By augmenting the active natriuretic peptides, neprilysin inhibition increases generation of myocardial cyclic guanosine 3′5′ mono phosphate, which improves myocardial relaxation and reduces hypertrophy.[^14^,^15^] However, the development of omapatrilat was discontinued because of an increased risk of angiooedema which caused by accumulation of bradykinin secondary to both neprilysin and ACE inhibition.^[16]^ Furthermore, few systematic studies demonstrating whether cardiac function is improved after sacubitril-valsartan therapy in patients with HF have been reported.

To determine the effects and safety of sacubitril-valsartan in patients with heart failure, we performed a systematic literature review and meta-analysis of randomized clinical trials (RCTs).

## 2 Materials and methods

### 2.1 Search strategy

This study was performed according to the Cochrane Handbook for Systematic Reviews of Interventions,^[17]^ and it published according to the Preferred Reporting Items for Systematic Reviews and Meta-Analyses Statement.^[18]^ The protocol was registered in Prospero database (registration number CRD42021281250).

We searched the following electronic databases for RCTs published no later than September 2020: Cochrane Library, PubMed web of science, CNKI, VIP, Medline, ISI Web of Science, CBMdisc, and Wanfang database. No limits were set on language. The search strategy included the following terms:

([“Heart failure” OR “Cardiac insufficiency” OR “left ventricular systolic dysfunction” OR “heart decompensation” OR “myocardial failure”] AND [“Sacubitril-Valsartan” OR “Angiotensin–Neprilysin Inhibition”] AND [“left ventricular ejection fraction” OR “LVEF”]).

### 2.2 Inclusion and exclusion criteria

The inclusion criteria for the selected studies were as follows: a) studies that measured left ventricular ejection fraction (LVEF) in patients with heart failure undergoing Sacubitril-Valsartan therapy as part of randomized controlled trials; b) studies that reported baseline and follow-up data on the mean and standard deviation of LVEF levels; c) studies included that LVEF < 40%; d) RCTs.

The exclusion criteria were as follows: a)Observational study; b) Animal research, c) research of other new drug intervention; d)The outcome indicators of literature application can not be extracted or calculated; e) The data were repeatedly published.

### 2.3 Data extraction and quality assessment

**Figure 1:**
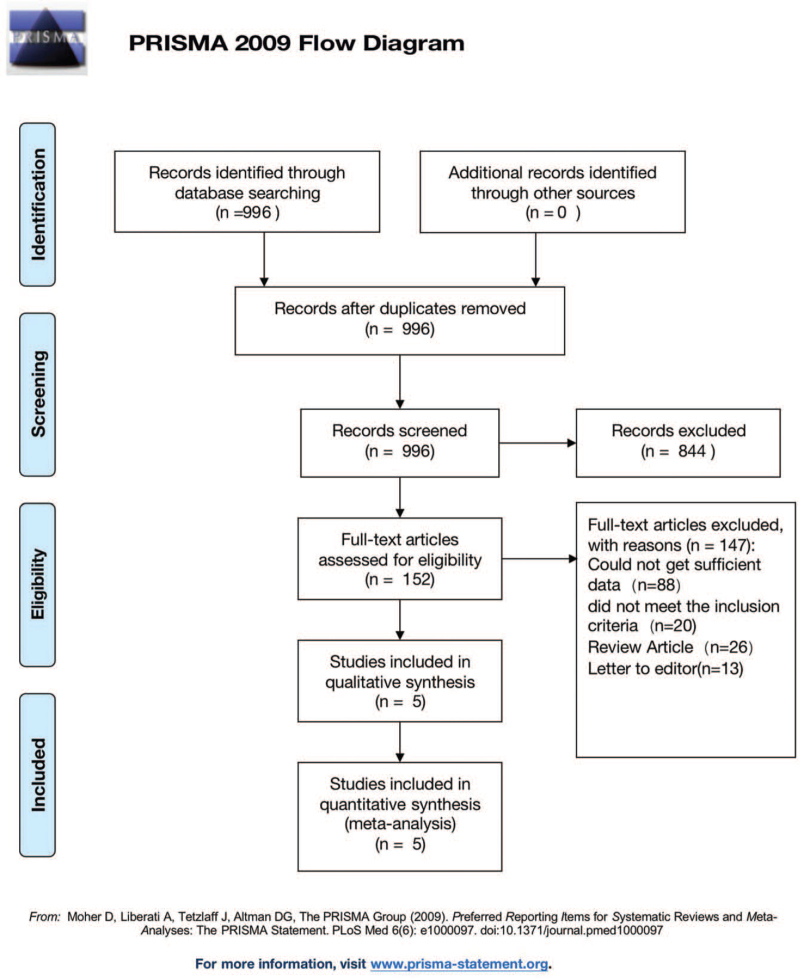
Flow diagram of literature search process.

**Figure 2:**
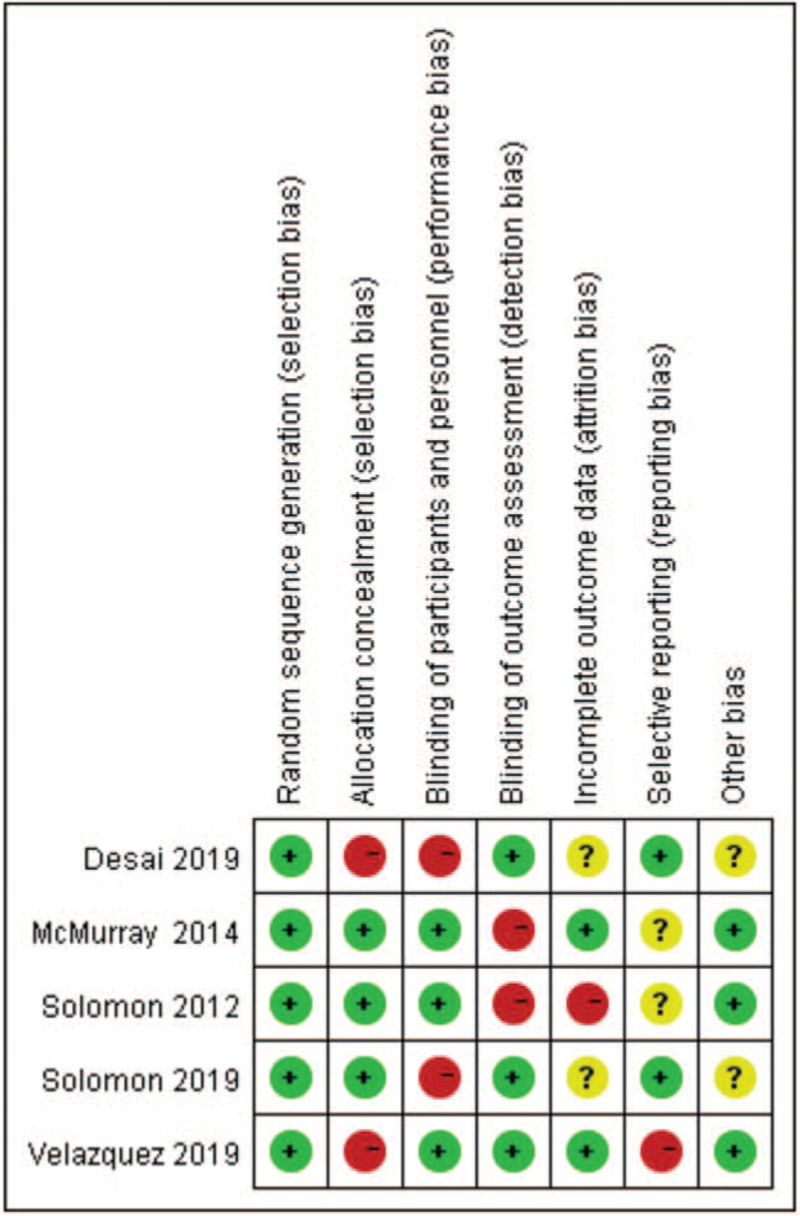
The risk of bias of randomized trials included in the meta-analysis.

Two researchers screened the study respectively, and checked the selected researches in accordance with the inclusion and exclusion criteria. When there was any objection to a certain research, the third researcher was consulted to finally determine the selected researches. The flow chart of literature screening is shown in Figure 1. Two researchers blindly collected the capital data (first author, year of publication, research method, research object, sample size, average age, course of treatment) and outcome indicators (echocardiographic indicators, mortality, rehospitalization rate due to heart failure, symptomatic hypotension, renal function injury rate, hyperkalemia, incidence of vascular edema). The bias risk assessment tool in Cochrane Handbook for systematic review of interventions (version 5.1.0) was used to evaluate the quality of the included studies. The results of the quality assessment are shown in Figure 2.

### 2.4 Statistical analysis

Review Manager Software (RevMan, version 5.2 from the Cochrane Collaboration) was used for data analysis and statistics of all outcome indicators. According to the heterogeneity test results, the effect model was determined. *I* ^2^ ≥ 50% indicates greater heterogeneity, and the random effect model (RE) was selected; *I* ^2^ ≤ 50% indicates that the heterogeneity is within the acceptable range, and the fixed effect model (FE) is selected. Continuous variables were combined with weighted mean difference (WMD), and binary variables were combined with RR. When *P* < .05, it was considered that there were significant differences in the changes of each outcome index. Subgroup analysis was used to identify the source of heterogeneity, and sensitivity analysis was used to assess the impact of individual studies on the overall results.

### 2.5 Ethical approval

Ethical approval was not necessary because our study was a meta-analysis, and which belonging to a form of secondary analysis.

## 3 Results

### 3.1 Flow chart of study selection

A total of 996 studies were identified in the initial literature search. A flow diagram of the study selection process is shown in Figure 1. As part of the initial screening of titles and abstracts, we excluded 996 citations, and the 152 articles were to be retrieved for full text review, 147 articles were excluded: 98 studies did not report baseline LVEF and/or outcomes related to cardiac function, 20 studies did not meet the inclusion criteria, and 26 studies were review articles, 13 studies were letter to editor. Therefore, 5 randomized, double-blind, controlled trials[^19^,^20^,^21^,^22^] were included in the meta-analysis: 3 RCT studies comparing sacubitril-valsartan with enalapril in patients with heart failure,[^19^,^13^,^20^] and 2 comparing sacubitril-valsartan with valsartan in patients with heart failure.[^21^,^22^]

### 3.2 Characteristics of included studies

The characteristics of included studies are summarized in Tables 1 and 2. Among the 5 studies eligible for the meta-analysis, a total of 14841 subjects were enrolled. Among them, 7414 subjects were randomized to receive sacubitril-valsartan. Five studies were conducted in Western countries. Five studies provide the mean age and standard deviation for each group of patients. The duration of therapy ranged from 2 to 27 months. Three RCT studies compared sacubitril-valsartan with enalapril in patients with heart failure,[^19^,^13^,^20^] and 2 compared sacubitril-valsartan with valsartan in patients with heart failure.[^22^,^22^] Figure 2 shows the risk of bias of randomized trials included in the meta-analysis. Randomization was performed according to a computer-generated random list or by means of a randomly generated number pattern in a majority of the trials.[^19^,^20^,^21^,^22^] The randomized trials included in our study were characterized by a low risk of incomplete outcome data and selective outcome reporting. Five randomized trials included in our study were characterized by a high risk of blinding of participants and personnel and outcome assessment.[^19^,^20^,^21^,^22^] Moreover, all randomized trials were with an unclear risk of other bias. In conclusion, the quality of these studies was moderate to high (Fig. 2).

**Table 1:** Characteristics of the 5 studies in the meta-analysis.

Author Year	Country	Age (EG vs CG) Mean ± SD	Size EG/CG	Types of studies and intervention	Doses	Therapy (months)
Desai 2019^[19]^	American	67.8 ± 9.8 vs66.7 ± 8.5	231/233	RCT comparing the use of sacubitril/valsartan (Expermental group) + enalapril (Control group)	Sacubitril/valsartan 97/103 mg twice	12
McMurray 2014^[20]^	UK	63.8 ± 11.5 vs63.8 ± 11.3	4187/4212	RCT comparing the use of sacubitril/valsartan (Expermental group) + enalapril (Control group)	Sacubitril/valsartan 200 mg twice	27
Velazquez 2019^[21]^	American	61.1 ± 1.2 vs63.2 ± 1.4	440/441	RCT comparing the use of sacubitril/valsartan (Expermental group) + enalapril (Control group)	Sacubitril/valsartan 97/103 mg twice	2
Solomon 2019^[22]^	UK	72.7 ± 8.3 vs72.8 ± 8.5	2407/2389	RCT comparing the use of sacubitril/valsartan (Expermental group) + valsartan (Control group)	Sacubitril/valsartan 97/103 mg twice	8
Solomon 2012^[23]^	UK	70.9 ± 1.6 vs71.2 ± 2.1	149/152	RCT comparing the use of sacubitril/valsartan (Expermental group) + valsartan (Control group)	Sacubitril/valsartan 200 mg twice	6

**Table 2:** Characteristics of the 5 included studies on LVEF.

				LVEF			
Author	Country	Age	Intervention	Pre-T	Post-T	Therapy months	Blinding
Desai 2019^[19]^	Western	≥18	EG: sacubitril/valsartan 97/103 mg twiceCG: enalapril 10 mg twice	34 ± 1033 ± 11	36 ± 1034 ± 9.3	<6	Double-blind
McMurray 2014^[20]^	Western	≥18	EG:sacubitril/valsartan 200 mg twiceCG: enalapril 10 mg twice	38.52 ± 3.136.91 ± 3	40 ± 2.838.9 ± 1.3	≥6	Double-blind
Velazquez 2019^[21]^	Western	≥18	EG: sacubitril/valsartan 97/103 mg twiceCG: enalapril 10 mg twice	34.14 ± 8.2634.08 ± 8.24	44.47 ± 5.4937.26 ± 6.79	<6	Double-blind
Solomon 2019^[22]^	Western	≥18	EG: sacubitril/valsartan 97/103 mg twiceCG: valsartan 160 mg twice	36.5 ± 12.6937.3 ± 15.54	48.2 ± 9.7043.8 ± 7.39	≥6	Double-blind
Solomon 2012^[23]^	Western	≥18	EG: sacubitril/valsartan 200 mg twiceCG: valsartan 160 mg twice	32.7 ± 10.4033.6 ± 14.70	58.3 ± 7.7058.1 ± 8	≥6	Double-blind

### 3.3 Pooled analysis

**Figure 3:**
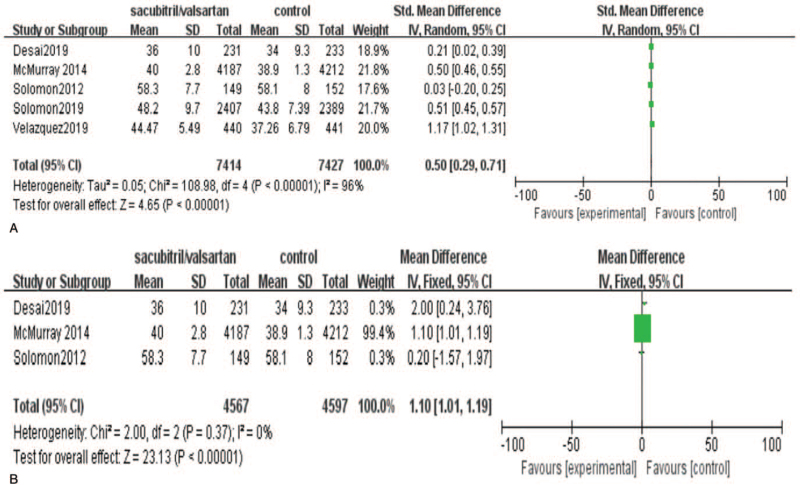
Meta-analysis on left ventricular ejection fraction (%) in the sacubitril/valsartan group versus Control group.

Meta-analysis of data from the 5 eligible studies[^19^,^20^,^21^,^22^] showed that left ventricular ejection fraction (LVEF) levels were significantly improved in patients with heart failure in the sacubitril-valsartan group (random effect model, standard mean differences [SMD] = 0.5, 95% CI = [0.29, 0.71]; Fig. 3A.). Considering heterogeneity existence (*I* ^2^ = 96% and *P* .00001; Fig. 3A), we underwent sensitivity analysis. We removal 2 studies[^21^,^22^] from the analysis, the results indicated that no heterogeneity was observed across studies (*I* ^2^ = 0 and *P* = .37; Fig. 3B), and it did not influence our primary analyses for LVEF (fixed-effects model, SMD = 1.1, 95% CI = [1.01, 1.19]; Fig. 3B.). From the analysis above, this 2 studies were the main reason for high heterogeneity which was also validated by the funnel plot (Fig. 9). Then we conducted a thorough read on the article, and the possible reasons are as follows. First, the studies could not rule out selection bias that patients were governed by specific characteristics which could influence results. Second, the size of Velazquez's study was small compared with other included studies.

**Figure 4:**
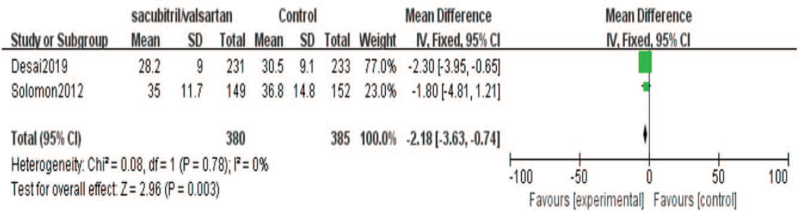
Meta-analysis of Left atrial volume index (LAVI) in patients with sacubitril/valsartan compared with ARB or ACE inhibitor.

Meanwhile, we conducted a forest plot for the meta-analysis of the effect of sacubitril-valsartan on left atrial volume index (LAVI). Two included studies[^19^,^22^] reported the results of LAVI. There were 380 cases in the sacubitril-valsartan group and 385 cases in the control group. The heterogeneity was low [*I* ^2^ = 0%, *P* = .78]. Meta-analysis showed that LAVI of sacubitril-valsartan group was lower than that in control group. The improvement of LAVI was more obvious after sacubitril-valsartan treatment, shown in Figure 4.

**Figure 5:**
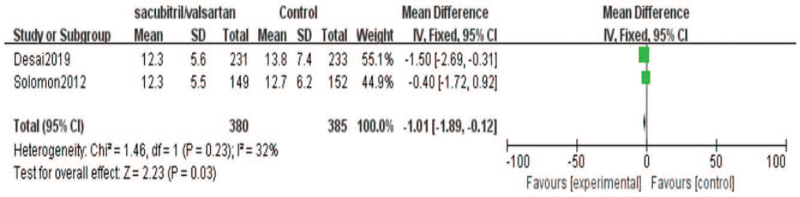
Meta-analysis of E/e in patients with sacubitril/valsartan compared with ARB or ACE inhibitor. Note: E/e: ratio of maximum filling velocity of early diastolic mitral valve to maximum velocity of early diastolic mitral annulus.

Furthermore, we did the meta-analysis of the effect of sacubitril-valsartan on ratio of maximum filling velocity of early diastolic mitral valve to maximum velocity of early diastolic mitral annulus (E/e). Two included studies[^19^,^22^] reported the results of E/e. There were 380 cases in the sacubitril-valsartan group and 385 cases in the control group. Meta-analysis showed that E/e of sacubitril-valsartan group was lower than that of control group. The improvement of E/e was more obvious after sacubitril-valsartan treatment (95% CI = [−1.89, −0.12], *P* = .03) (Fig. 5).

**Figure 6:**
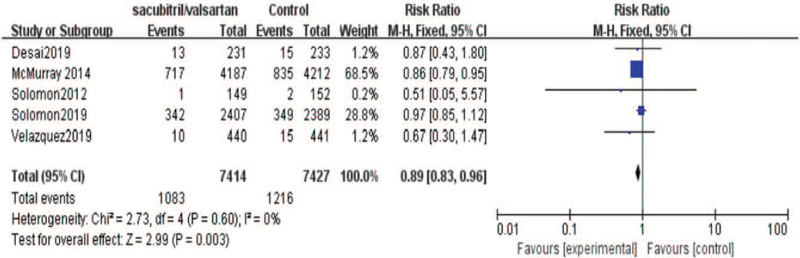
Meta-analysis of cardiovascular death in patients with sacubitril/valsartan compared with ARB or ACE inhibitor.

**Figure 7:**
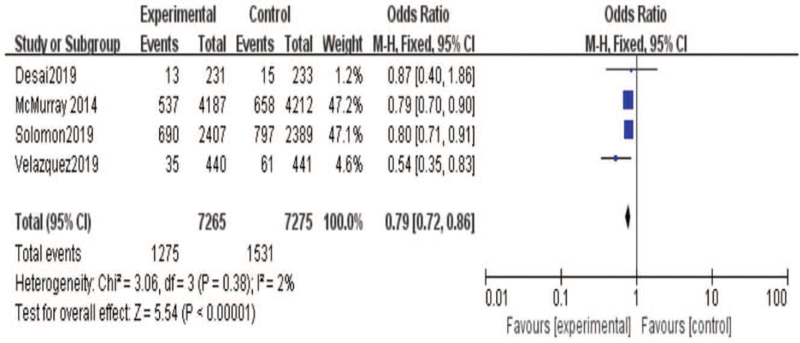
Meta-analysis of rehospitalization rate in patients with sacubitril/valsartan compared with ARB or ACE inhibitor.

**Figure 8:**
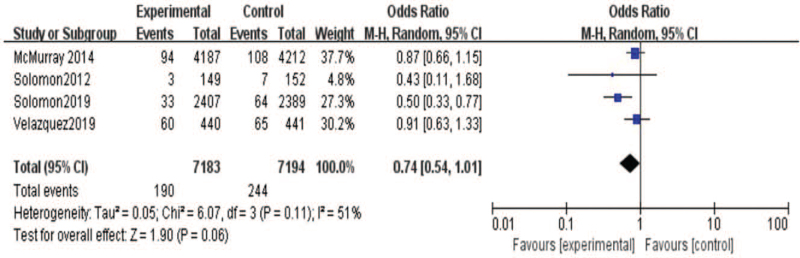
Meta-analysis of renal function in patients with sacubitril/valsartan compared with ARB or ACE inhibitor.

On the other hand, we research the effect of sacubitril-valsartan on cardiovascular death. As shown in the Figure 6, 5 included studies[^19^,^20^,^21^,^22^] reported the results of cardiovascular death. There were 7414 patients in the sacubitril-valsartan group and 7427 populations in the control group. Meta-analysis showed that cardiovascular death of sacubitril-valsartan group was lower than that of control group (95% CI = [0.83,0.96], *P* = .003). Moreover, we also analysis the rehospitalization rate between 2 groups. As shown in the Figure 7, 4 included studies[^19^,^20^,^21^] reported the results of rehospitalization rate. There were 7265 patients in the sacubitril-valsartan group and 7275 patients in the control group. The results demonstrated that rehospitalization rate of sacubitril-valsartan group was obvious improvement than that of control group (95% CI = [0.72, 0.86], *P* < .00001). Besides, we conducted the renal function between 2 groups after treatments. There were 4 included studies[^13^,^14^,^15^,^16^,^17^,^18^,^19^,^20^,^21^,^22^] reported the condition of renal function. The analysis showed that renal function was no significant difference between 2 group (95% CI = [0.54, 1.01], *P* = .06)(Fig. 8).

### 3.4 Sensitivity analysis and publication bias

**Figure 9:**
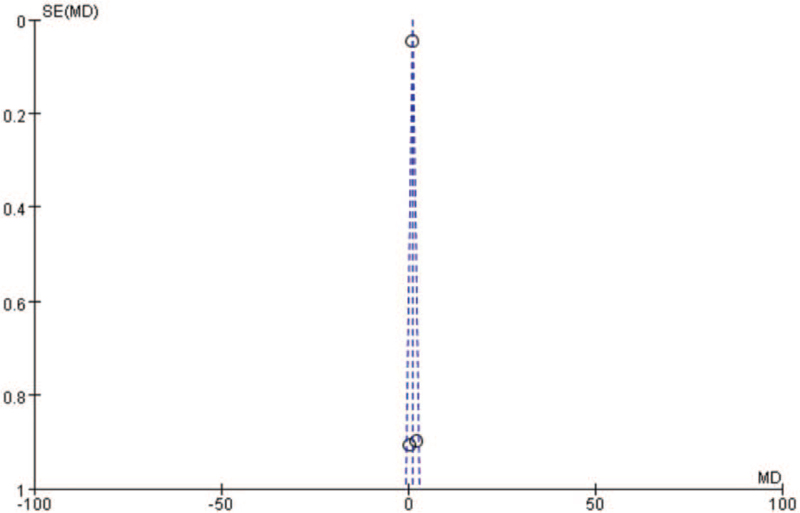
The funnel plot of data in the analysis of LVEF.

Sensitivity analysis revealed that removal of any 1 study from the analysis did not subvert the results of the pooled analysis (SMD = 0.5, 95% CI = [0.29, 0.71, *P* < .00001). We removal 2 studies[^19^,^22^] from the analysis, the results indicated that no heterogeneity was observed across studies (*I* ^2^ = 0 and *P* = .37; Fig. 3B), and it did not influence our primary analyses for LVEF (fixed-effects model, SMD = 1.1, 95% CI = [1.01, 1.19]; Fig. 3B.). Therefore, the outcome of the pooled analysis can be regarded with a higher degree of certainty. Furthermore, we constructed funnel plots to evaluate publication bias. The funnel plots (Fig. 9) for LVEF showed no publication bias.

## 4 Discussion

The present meta-analysis demonstrated that left ventricular ejection fraction (LVEF) was improved after sacubitril-valsartan in patients with heart failure. Combined outcome indicators showed that, combined outcome indicators showed that, compared with control group, the left ventricular volume index (LAVI), the E/e’ (*P* < .05), the cardiovascular death, and the rehospitalization rate of heart failure decreased more significantly, but it had no effect on renal function.

Kang et al^[23]^ did a meta-analysis about sacubitril/valsartan in patients with heart failure and chronic kidney disease, they found that sacubitril/valsartan significantly increased estimated glomerular filtration rate (eGFR, MD = 1.90, 95% CI [0.30, 3.50], *P* = .02), which was partly consistent with our results, however, this study did not conduct the effect on cardiac function of sacubitril/valsartan. On the other hand, Nielsen's^[24]^ study demonstrated that sacubitril/valsartan compared with control decreases the risk of death, risk of serious adverse events, risk of hospi-talizations and NT-proBNP, and it might be beneficial for patients with HFrEF, which was partly consistent with our results, nevertheless, this study mainly studied the patients with HFrEF and it only compared NT-proBNP, which had difference with our study. In our study, the results showed that compared with enalapril and valsartan, sacubitril-valsartan had more significant improvement in LVEF and cardiac function. Undeniably, Zhang et al^[25]^ had suggested that sacubitril/valsartan significantly decreased the risk of death from all causes or cardiovascular causes in HF, which is consistently with our study. On the other hand, we did more indexes such as ventricular volume index (LAVI), the E/e’, rehospitalization rate and left ventricular ejection fraction to compare the efficacy and safety of sacubitril/valsartan In heart failure participants, meta-analysis showed that sacubitril/valsartan could ameliorate cardiovascular death, rehospitalization rate. Sacubitril/valsartan could benefit for patients with heart failure, thus, our meta-analysis is more comprehensive conclusion.

The main pathogenesis of heart failure is related to renin angiotensin aldosterone system (RASS), sympathetic nervous system (SNS) and natriuretic peptide system (NPS).[^26^,^27^,^28^,^29^,^30^] In the early stage of the disease, the activation of RAAS and SNS can play a compensatory role in the heart. However, if they are activated continuously for a long time, they will promote the necrosis of myocardial cells, induce ventricular remodeling, and further progress and deterioration of cardiac function until death.^[31]^ Sacubitril-valsartan can also inhibit the activation of RAAS system, enkephalinase and the degradation of natriuretic peptide.[^32^,^33^] Sacubitril-valsartan should augment this endogenous defence mechanism and could be beneficial in heart failure with both reduced and preserved ejection fraction.^[34]^

The strength of the present meta-analysis is that it is the first comprehensive review to summarize the available evidence for assessing the effects and safety of sacubitril-valsartan in patients with HF. In addition, the results are stronger than any single study given that the included RCTs demonstrate homogeneity. We are plausible biological mechanisms to explain the cardioprotective effect of angiotensin-Neprilysin inhibition. We did not detect significant heterogeneity or publication bias. Based on these factors, this review should provide convincing evidence regarding the cardioprotective effect of sacubitril-valsartan in patients with HF.

The present meta-analysis also has some weakness. The primary limitation is the limited number of studies analyzed. We only included 5 studies, and it could not conduct a meta-regression analysis. In addition, we did not analyze the severity of heart failure in subgroup. Moreover, other measurements such as smoking status, obesity, and other lifestyle factors should be considered confounding factors, because the results of our study were based on unadjusted estimates. Finally, this review included small sample-size, single-center studies with clinical heterogeneity and variable patient backgrounds, which could have resulted in low statistical power and inconsistent results among studies. Therefore, large sample-size clinical trials should be carried out to further verify the effects and safety of Angiotensin-Neprilysin inhibition in patients with HF.

## 5 Conclusion

In conclusion, this review represents a comprehensive analysis of the assessment the effects and safety of sacubitril-valsartan treatment in patients with HF and includes only RCTs. It showed that there was significant improvement of LVEF after sacubitril-valsartan treatment in patients with HF. Furthermore, there was no impact on renal function. The data suggest that sacubitril-valsartan may ameliorate cardiac function in HF disease. Additional studies are required to further verify the effects and safety of sacubitril-valsartan in patients with HF. Considering the limited number of studies analyzed, large sample-size clinical trials are necessary to verify the long-term effects of Angiotensin-Neprilysin inhibition on cardiac function in HF.

## Author contributions

**Data curation:** Jianyi Zhou.

**Methodology:** Guiting Xie.

**Software:** Guiting Xie.

**Validation:** Jiezhong Lin.

**Visualization:** Jiezhong Lin.

**Writing – original draft:** Jiezhong Lin, Jianyi Zhou.

**Writing – review & editing:** Jinguang Liu.
